# More cost-effective management of patients with musculoskeletal disorders in primary care after direct triaging to physiotherapists for initial assessment compared to initial general practitioner assessment

**DOI:** 10.1186/s12891-019-2553-9

**Published:** 2019-05-01

**Authors:** Lena Bornhöft, Jörgen Thorn, Mikael Svensson, Lena Nordeman, Robert Eggertsen, Maria E. H. Larsson

**Affiliations:** 1 Department of Health and Rehabilitation, Institute of Neuroscience and Physiology, Sahlgrenska Academy, University of Gothenburg, Gothenburg, Sweden; 2 Närhälsan Torslanda Rehabilitation Centre, Gothenburg, Sweden; 3 Department of Public Health and Community Medicine, Institute of Medicine, Sahlgrenska Academy, University of Gothenburg, Gothenburg, Sweden; 4 Närhälsan Research and Development Primary Health Care, Region Västra Götaland, Sweden

**Keywords:** Triage, Primary care, Physiotherapy, Musculoskeletal disorders, Cost-benefit analysis

## Abstract

**Background:**

A model for triaging patients in primary care to provide immediate contact with the most appropriate profession to treat the condition in question has been developed and implemented in parts of Sweden. Direct triaging of patients with musculoskeletal disorders (MSD) to physiotherapists at primary healthcare centres has been proposed as an alternative to initial assessment by general practitioners (GPs) and has been shown to have many positive effects. The aim of this study was to evaluate the cost-effectiveness from the societal perspective of this new care-pathway through primary care regarding triaging patients with MSD to initial assessment by physiotherapists compared to standard practice with initial GP assessment.

**Methods:**

Nurse-assessed patients with MSD (*N* = 55) were randomised to initial assessment and treatment with either physiotherapists or GPs and were followed for 1 year regarding health-related quality of life, utilization of healthcare resources and absence from work for MSD. Quality-adjusted life-years (QALYs) were calculated based on EQ5D measured at 5 time-points. Costs for healthcare resources and production loss were compiled. Incremental cost-effectiveness ratios (ICERS) were calculated. Multiple imputation was used to compensate for missing values and bootstrapping to handle uncertainty. A cost-effectiveness plane and a cost-effectiveness acceptability curve were construed to describe the results.

**Results:**

The group who were allocated to initial assessment by physiotherapists had slightly larger gains in QALYs at lower total costs. At a willingness-to-pay threshold of 20,000 €, the likelihood that the intervention was cost-effective from a societal perspective including production loss due to MSD was 85% increasing to 93% at higher thresholds. When only healthcare costs were considered, triaging to physiotherapists was still less costly in relation to health improvements than standard praxis.

**Conclusion:**

From the societal perspective, this small study indicated that triaging directly to physiotherapists in primary care has a high likelihood of being cost-effective. However, further larger randomised trials will be necessary to corroborate these findings.

**Trial registration:**

ClinicalTrials.gov NCT02218749. Registered August 18, 2014.

## Background

Musculoskeletal disorders (MSDs) are one of the most prevalent groups of debilitating health conditions found globally [1]. The global costs incurred because of MSD are enormous. Healthcare expenditure constitutes a substantial portion of these costs. Wieser et al. found that 13,4% of healthcare expenditure in Switzerland was spent on musculoskeletal conditions [2]. These conditions are, usually, first seen and treated within primary care. MSD accounts for 14–17% of all primary care visits [3, 4]. Treatment costs for those musculoskeletal conditions seen within primary care consist of direct costs mainly for consultations with various healthcare professionals within primary care and rehabilitation services, costs for radiological examinations, for consultations with specialists within secondary care, as well as medication.

The largest factor affecting the cost of MSD for society is, however, production loss [5]. MSDs are common among the working age population and are the direct cause for a considerable level of short-term and long-term sick-leave, as well as work absence for healthcare visits, leading to production loss and costs for the community at large [2, 6]. The economic impact of production loss due to MSD has been estimated to be up to 2% of the gross domestic product in Europe [7].

Standard praxis for the management of MSD in primary care is for a GP to make an initial assessment and provide first-line treatment, referring to other care-givers as necessary [8]. Inadequacies in current management have been reported which affect health resource use [9, 10] and may even affect clinical course for MSD [11]. GPs frequently refer to physiotherapists in their management of MSDs [12–14]. Physiotherapists have been shown to have adequate competence regarding the treatment of many kinds of musculoskeletal conditions and as initial care-giver for such conditions [15–18]. Observational studies based on medical records have shown that, when physiotherapists are initial care-givers, savings may be made in terms of healthcare resources such as visits to GPs, prescriptions, and referrals for radiological examinations and specialist assessments [19–21]. It is possible that it may be viable and cost-effective for physiotherapists to take over responsibility for many of the assessments of MSD now made by GPs.

In western Sweden, a model for triaging patients in primary care has been implemented over the last several years with good effect [22]. The model is adapted from triage systems used in emergency care to conditions and patients commonly seen in primary care [22, 23]. For conditions not requiring immediate GP assessment or treatment, the triage model aims to provide immediate contact with other appropriate professions. According to this model, most patients seeking help for MSD are triaged by nurses directly to physiotherapists for initial examination and treatment, reducing the burden on GPs and likely leading to a different clinical course [11, 19]. The triage model has been evaluated from the organisational perspective and has been shown to have many advantages, such as increased access, improved work environment, and good patient satisfaction, without leading to any serious adverse events [22]. Other triage models for MSD have also been shown to have a myriad of positive effects [24–26].

Our study group recently conducted a randomised controlled trial (RCT) comparing the effects on patients’ health over time after an initial consultation with either a physiotherapist or a GP. This indicated that initial triaging of patients with MSD directly to physiotherapists led to higher health-related quality of life and at least as good effect on pain, musculoskeletal function and risk for developing chronic symptoms as an initial consultation with a GP [11]. However, no studies have been found which examine the costs of triaging to physiotherapists in relation to health effects.

The present health economic study is based on the fore-mentioned RCT [11] and aims to evaluate the cost-effectiveness from the societal perspective of this new care-pathway through primary care regarding triaging patients with MSD to initial assessment by physiotherapists compared to standard practice with initial GP assessment.

## Methods

### Study population and setting

The study population and setting have been described in more detail earlier [11]. Participants were recruited from three Swedish primary care clinics with registered patients from varying sociodemographic levels, such as are representative for the urban Swedish population. These clinics all had several years’ experience working according to the triage model described above. Patients with MSD were first assessed by triage nurses as suitable for triaging directly to physiotherapists. Clinical triage instructions were to book all patients seeking help for MSD and with no apparent immediate need for GP services directly to a physiotherapist [22]. Focus was to be placed on MSDs with recent debut or recent flare-up. Other inclusion criteria were being of working age (16–67 years) and having sufficient command of either Swedish or English to fill out the questionnaires. Patients who required home visits or primarily needed medical aids were excluded. Patients who had ongoing treatment for the current MSD were not eligible, nor were those seeking help for chronic conditions with unchanged symptoms the last three months and who had already tried physiotherapeutic treatment for this condition. The inclusion and exclusion criteria were formulated to mirror as accurately as possible the patients which the nurses normally triaged directly to physiotherapists, to make it easier for them to follow the study protocol and to ensure that study results would be representative for the clinical setting. Participants received no financial or other compensation.

### Study design

The intervention consisted only of determining which profession would make the initial assessment. All visits and services after the initial visit were treatment as usual. Eligible patients agreeing to participate in the study were randomised to a first visit with either a physiotherapist (intervention) or a GP (treatment as usual (TAU)). The healthcare providers were unaware of study participation and were free to provide the treatment and services they thought appropriate. Participants were followed for 1 year from inclusion regarding health-related quality of life, healthcare resource use and absence from work for MSD. A cost-efficiency analysis was performed from a societal perspective where formal and informal healthcare costs as well as production effects for one year following inclusion were compared for the group initially assessed by a physiotherapist and the group initially assessed by a GP. As the relevant time period was 1 year, no discounting was necessary. A power analysis was performed for the original RCT requiring 63 participants per group [11]. Recruiting was, however, discontinued early when impending organizational changes threatened to hinder study conditions with physiotherapist placement at the primary healthcare centres.

### Health outcomes

Health-related quality of life (HRQoL) was chosen as the health benefit in the evaluation as a generic measure of health improvement. This was measured at inclusion and at 2, 12, 26 and 52 weeks with Euroqol 5 dimensions-3 L (EQ5D) [27]. Questionnaires were sent home to participants with reminders as necessary. Quality-adjusted life-years (QALYs) were calculated based on index values for EQ5D, assessed using the Dolan tariff, and the relevant time after inclusion [28]. QALYs were calculated for each participant based on linear interpolation between each measurement point and then combining the “areas under the curve”. Regression analysis was used to adjust for baseline differences in HRQoL [29].

### Costing

The participants were given paper diaries to record healthcare services and visits and were instructed to record only services and visits which were relevant for the MSD for which they were included in the study. Diaries were collected and replaced at each follow-up time together with EQ5D. Healthcare resources in the form of visits to GPs and physiotherapists, referrals to secondary care specialists and to radiological examinations, prescription details and the number of days of sick-leave were reported. Data from the diaries was supplemented, when available, by information in the medical records.

Formal healthcare costs for GP and physiotherapist visits and referred services were collected from the healthcare organisation. Capital and operating costs and social fees were included in mean hourly rates each for physiotherapists and GPs, which were then used to calculate total costs for each participant’s visits. Prescribed pain medication was recorded per patient during the follow-up period and then linked to Swedish market prices.

There are several accepted methods used to determine how to value production costs in cost-effectiveness analyses [30]. Here, the human capital approach was used to value production loss [30, 31]. This includes all work hours lost due to health problems and healthcare treatment. Informal costs for patients’ time use and production loss due to healthcare visits and sick-leave during the follow-up period were based on mean gross wages (including social fees) in the area. The number of sick-leave days was self-reported, double-checked in the medical records and includes the first week of absence from work which, in Sweden, does not require a physician’s sick-note. Production loss for visits to healthcare providers was based on a presumed 30-min visiting time for GPs, 45 min for physiotherapists, 1 h for referred visits and examinations plus 1-h travel/waiting time for all visits. If the patient was on sick-leave at the time of the visit, then compensation level for unpaid work with net mean wage after tax was used.

### Measures of cost-effectiveness

The incremental cost-effectiveness ratio (ICER) was calculated as the ratio between the difference in mean costs and mean QALYs (*∆Cost*/*∆QALYs*) between the physiotherapist group and the TAU group from randomisation to the 12-month follow-up. The ICER can be interpreted as the *price tag* per one-unit change of mean QALYs [32]. Multiple imputation (MI) was used to deal with missing data. A multivariate MI approach with 20 sequential imputations based on chained regressions using Stata v.15 was implemented, with baseline HRQoL, age, sex, comorbidities and treatment status used as factors.

Sampling uncertainty was assessed using non-parametric bootstrapping, where ICERs were estimated based on 1000 bootstrap resamples and summarised in a cost-effectiveness plane (CE-plane) and in a cost-effectiveness acceptability curve (CEAC).

The CE-plane is typically divided into four quadrants (north-east, north-west, south-east, and south-west). The south-east quadrant represents a situation where the intervention dominates over the control leading to both health improvements and monetary savings. The north-east and south-west quadrants represent the “trade-off” quadrants where increases in costs must be compared to improvements in health (north-east quadrant) or cost-savings to reduced health (south-west quadrant). Lastly, the north-west quadrant represents a situation where the intervention is dominated by the control leading to reduced health at greater costs.

The CEAC shows the probability that the intervention is cost-effective compared to the control for a range of different maximum acceptable ICERs. This is a useful illustration considering that the de facto “threshold value” (or willingness-to-pay per QALY) differs between different healthcare contexts and jurisdictions.

## Results

Detailed demographic statistics describing the participants have been published earlier [11]. There were no significant differences between the intervention and TAU groups at baseline regarding age, sex, comorbidities, depression or country of birth. There were 28 patients in the intervention group and 27 in the TAU group. The spread of MSDs was similar in both groups. Consent to use information in the medical records was provided by 21 of the participants.

All 8 patients with recorded sick-leave and 7 out of 8 patients with recorded referrals had adequately completed their diaries or provided consent to use the medical records. A significant difference between groups regarding number of patients who received referrals for radiological examinations and assessments by specialists within secondary care was found (*p* = 0.019) (Table 1). Otherwise, there were no significant differences for number of patients receiving prescriptions, sick-notes or having consultations with either physiotherapists or GPs after the initial triage visit.

**Table 1:** Healthcare services for intervention and control groups

	Intervention *N* = 27	TAU *N* = 26	*P*-value
Physiotherapist, n (%)	12 (44)	10 (38)	0.649
GP, n (%)	7 (26)	4 (15)	0.242
Referrals, n (%)	1 (4)	7 (27)	**0.019**
Prescriptions, n (%)	7 (26)	9 (35)	0.516
Sick-notes, n (%)	4 (15)	4 (15)	1.000

*Intervention = Initially triaged to physiotherapists. TAU = Treatment as usual (initially triaged to GPs). Physiotherapist and GP = number of cases with visits after the triage visit. Referrals, prescriptions, sick-notes = number of cases receiving MSD-related referrals, prescriptions or sick-notes from GPs during 1 year from the triage visit*

Significant values shown in boldface

Details of costs for each group are given in Table 2. All costs are expressed in Euros (€) at the relevant price levels for the study years 2014–2017 (Table 2).

**Table 2:** Total MSD-related healthcare costs for intervention and TAU groups

	Cost (€) (SD)		Unit price range	Δ (€)	*p*-value
	Intervention	TAU			
Healthcare resources					
Physiotherapist	4394 (232)	1891 (166)	44–45 €/hour	2503	0.109
GP	1673 (129)	3380 (120)	88–94 €/hour	− 1707	0.053
Referrals	135 (26)	1204 (105)	37–309 €	− 1069	0.062
Medication	261 (29)	168 (11)	5–14 €/drug	93	0.588
Total healthcare cost	6463 (388)	6643 (335)		− 180	0.872
Societal resources					
Production loss	46,659 (6392)	86,412 (12145)		−39,753	0.555
Unpaid work compensation	1567 (112)	1106 (100)		461	0.597
Total societal costs	48,226 (6449)	87,517 (12227)		−39,291	0.562
Total costs	54,690 (6644)	94,160 (12491)		−39,370	0.567

*Intervention = Initially triaged to physiotherapists. TAU = Treatment as usual (initially triaged to GPs). Physiotherapist = Time for physiotherapist visits x mean hourly rate. GP = Time for GP visits x mean hourly rate. Referrals = cost for radiological examinations plus time for specialist consultations in secondary care x mean hourly rate. Medication = cost for prescribed medications. Production loss = Time absent from work because of MSD-related sick-leave and healthcare visits x mean gross wages. Unpaid work compensation = Time for healthcare visits not requiring absence from work x mean net wages*

### Missing data

Of the 55 included patients, 2 were dropped because they were missing EQ5D data at baseline as well as at all subsequent measurement points. Among the remaining 53 patients, data was missing for costs and EQ5D between 0% (EQ5D at baseline) and 56% (EQ5D at the final measurement). There was no statistically significant difference between the intervention and TAU groups with respect to missing cost data (*p*-value = 0.17) or missing EQ5D data (*p*-values ranging from 0.15 to 0.7). Missing data was slightly more common in younger patients, but not related to the sex, country of birth or comorbidities.

### Cost-effectiveness

Table 3 shows the difference in mean costs, mean QALYs and the incremental cost-effectiveness ratio for the intervention compared to TAU. The point estimates show a lower cost as well as a higher number of QALYs in the intervention group compared to TAU, both from a societal perspective including healthcare costs, production loss and patient’s time costs as well as from a healthcare perspective including only formal healthcare costs. However, the differences in mean cost and QALYs are not statistically significant using the standard rule of thumb with 95% confidence intervals. The point estimates imply that the ICER represents an intervention that dominates care as usual, i.e. less expensive and better.

**Table 3:** Difference in mean costs, mean QALYs, and results for the ICER

Intervention vs TAU	Difference in mean cost (€) (95% CI)	Difference in mean QALYs (95% CI)	ICER
Societal perspective	− 3600 (−11,620 to 5540)	0.07 (−0.10 to 0.23)	Intervention dominates
Healthcare perspective	−30 (− 340 to 280)		Intervention dominates

*Results based on 53 patients with multivariate multiple imputation using chained regression equations where age, sex, comorbidities, treatment status, and baseline health status are used as factors. Intervention = Initially triaged to physiotherapists. TAU = Treatment as usual (initially triaged to GPs). € = Euros*

**Fig. 1:**
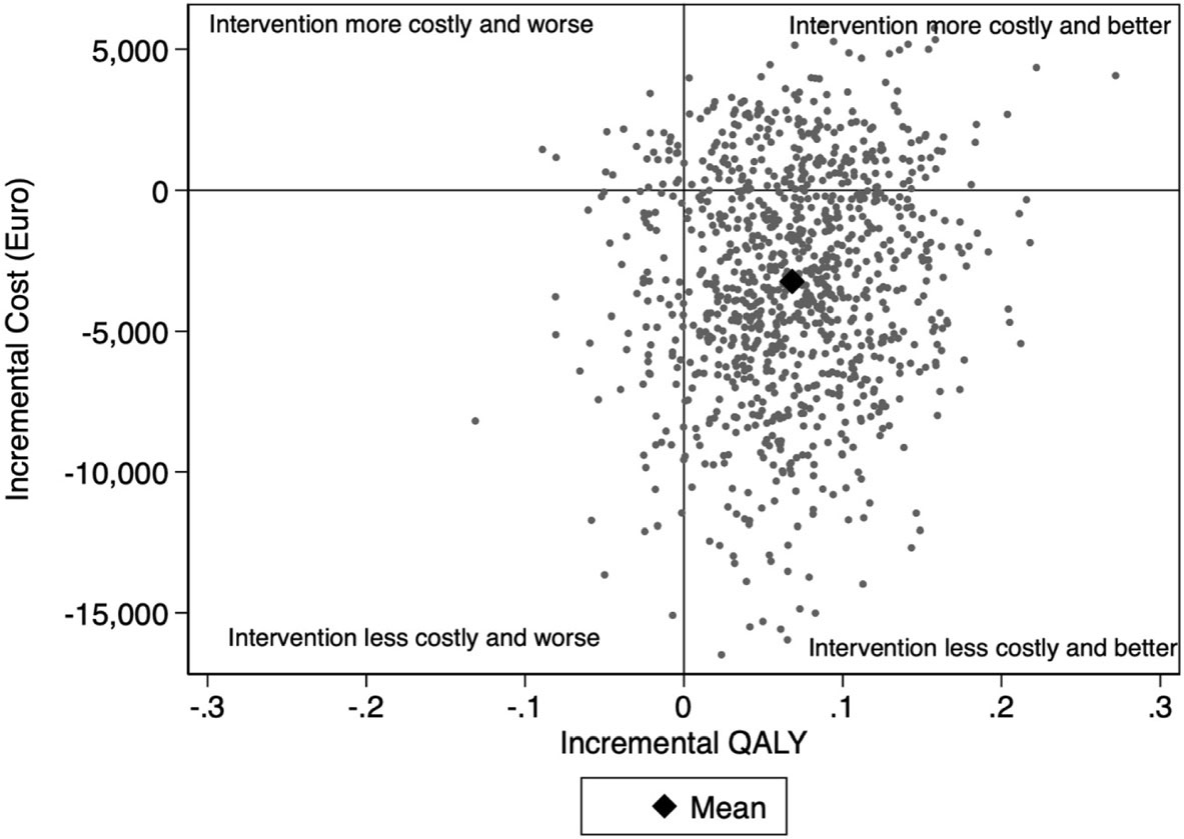
Bootstrapped ICERs in the cost-effectiveness plane

In Fig. 1, the uncertainty of the ICER is assessed based on 1000 bootstrapped replicates plotted on the CE-plane. The point estimate of the ICER (Table 3) is shown in the south-east quadrant (black diamond). This and the large mass of replicated ICERs in this quadrant represent a situation where the intervention dominates TAU (less costly and higher QALYs). A smaller share of the bootstrapped ICERs are located in the “trade-off” north-east (higher QALYs, higher costs) and south-west (lower QALYs, lower costs) quadrants. Finally, a small share of ICERs (7–8%) are also scattered in the north-west quadrant, representing a situation where the intervention is dominated by treatment as usual.

**Fig. 2:**
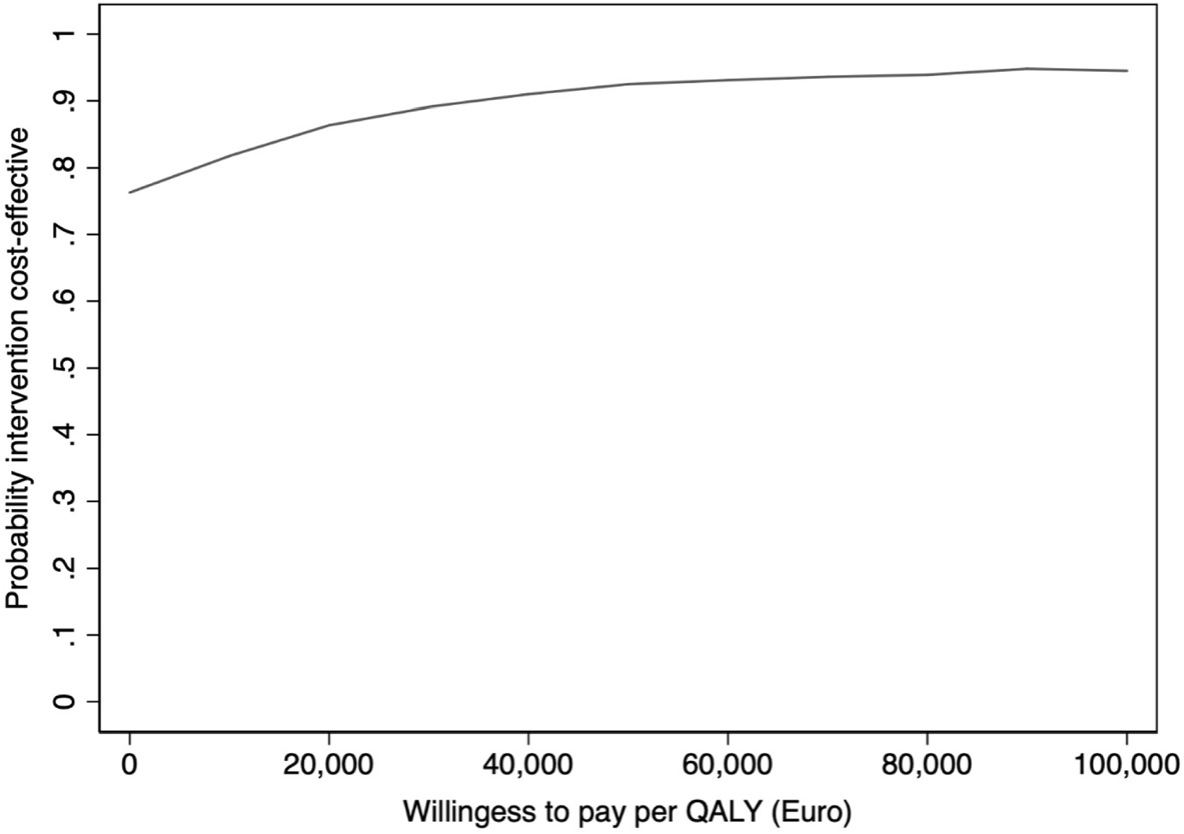
Cost-effectiveness acceptability curve based on 1000 bootstrapped ICERs

The cost-effectiveness acceptability curve (CEAC) shows the probability that the intervention is cost-effective compared to TAU for a range of different maximum acceptable ICERs (Fig. 2). At a willingness-to-pay threshold of 20,000 €, the probability that the intervention is cost-effective is approximately 85%. The probability that the intervention is cost-effective never reaches higher than 92–93% irrespective of the willingness-to-pay threshold because there is a small probability (approximately 7–8%), as seen in Fig. 1, that the intervention is dominated by TAU.

## Discussion

This cost-effectiveness analysis has led to a positive result in favour of the intervention with direct triaging to physiotherapists showing a high probability from a societal perspective of both higher level of QALYs and lower overall costs compared to standard primary care management of musculoskeletal disorders with initial consultation with a GP and later or no contact with a physiotherapist.

### Discussion of results

Patients who were triaged to a physiotherapist had generally higher costs for physiotherapy and lower costs for GP visits and referrals. The difference between groups in health outcomes was small but nonetheless favoured the physiotherapy-triage group. This combined with reduced sick-leave and production loss for the physiotherapist-triaged group led to the assessment of high probability of cost-efficiency. The difference in production loss was the single largest factor affecting cost-efficiency. It is possible that immediate active treatment of MSD speeds recovery, leading to less time off work. The RCT on which this economic evaluation was based indicated tendencies to better clinical course after initial triaging to physiotherapists [11].

Fewer patients who initiate treatment with a physiotherapist received referrals for radiological investigations and specialist assessments. It is known that, for prevalent sub-groups of MSDs such as low back pain and early osteoarthritis, excessive and unnecessary referrals are common and that patients are often active in requesting these services [33–35]. As physiotherapists do not usually have the right to make direct referrals, it requires an extra step, after a triage visit, for the patient to acquire a referral. If the information and treatment from the physiotherapist have already fulfilled many of the patient’s needs and reduced worry about long-term effects, it may affect the number of patients who feel it necessary to continue the process involved to acquire a referral.

Few management-based interventions are studied extensively. It is not uncommon to implement decisions and reorganise healthcare based only on presumed or calculated advantages for the healthcare organisation. This triage model has been studied from several perspectives and has not been found to lead to any disadvantages. The effects on health aspects, healthcare utilization, accessibility, work environment, patient satisfaction and now cost-efficiency all favour triaging to physiotherapist over traditional management [11, 19, 20, 22]. Involved stakeholders – patients, care-givers, the healthcare organisation and the community – all seem to win by reversing the order of involved care-givers. The results concerning the cost-efficiency of direct triaging to physiotherapist are corroborated by those few studies which have, in some way, compared the economic effects of primary physiotherapist and GP contacts in primary care [19–21, 36].

Changing standard praxis is never easy. However, the potential gains for doing so likely compensate for any difficulties. Continuing with primary GP contact for all conditions is less efficient [24] and often difficult to provide [37]. Enabling patients to consult physiotherapists directly on their own is not the same as actively triaging suitable patients to physiotherapists. Experience in Austria has indicated that unregulated access to healthcare leads to overutilization of predominantly the most specialized care as patients tend to prefer the care level they deem to have the highest competence but leading to an inefficient use of professional competence [38]. This study showed that, when the GP is the primary contact for patients with MSDs assessed as suitable for primary physiotherapeutic contact, less than 40% of patients actually continued on to see a physiotherapist. This may indicate that primary GP consultation not only delays (comparing referral to physiotherapist with direct triaging to physiotherapist) but often replaces active physiotherapeutic treatment which may be necessary for the most optimal long-term results [39]. It is known that early active treatment leads to advantages regarding clinical course and need for care for low back pain, which is the largest sub-group of MSDs [40–42]. Primary physiotherapeutic management of MSD may also lead to reduced focus on passive management aspects such as medication and sick-leave and to greater focus on self-responsibility and secondary prevention [39].

It seems that the triage nurses select relatively healthy patients for triaging to physiotherapists as the levels of sick-leave, medication and comorbidity are fairly low in both groups. It may be that the results of this study are most applicable to those patients with uncomplicated MSDs. This sub-group of patients likely has less need of GP services. However, even this sub-group needs optimal management to prevent development of chronic symptoms. This study also included patients who were in need of treatment by both GPs and physiotherapists. The favourable outcome applies to them as well. The order in which patients are assessed by different professions does seem to affect both clinical course and resource use.

### Methodological considerations

There was a considerable level of missing data in the study which was dealt with using multiple imputation. This is the best-practice recommended approach for dealing with missing data in cost-effectiveness analyses, as well as in other forms of applied statistical analyses of health data [43].

There are divided opinions about the most appropriate perspective to use in cost-effectiveness analyses [44]. Here, the focus was on the working-age population and on non-fatal conditions. It was, therefore, important to include societal costs for production loss and the human capitol approach was chosen to value production loss. For illnesses causing primarily short-term or no sick-leave, there is relatively little difference between the predominant methods to value production loss (human capitol approach and friction cost approach) [31]. A more restricted healthcare perspective and the associated ICER have also been presented for comparative purposes, indicating that triaging to physiotherapists leads to economic savings for the healthcare organization.

There are few and relatively small studies, with primarily observational study designs, investigating the effects of triaging to physiotherapists in primary care on resource use [19–21, 25]. This study had a robust randomised design adapted pragmatically to the clinical environment but was based on a small population. The study was underpowered due to practical difficulties, which should theoretically increase the risk for type II errors. A type II error means accepting the null-hypothesis (that there are no differences between groups) when the null-hypothesis is false. In other words, an underpowered study will increase the risk for underestimating the effects of the intervention. However, a significant difference between groups for referral frequency was found despite the small group size. Non-parametric bootstrapping was employed to reduce sampling uncertainty regarding cost-effectiveness. This statistical method has been found to provide accurate estimates of the mean even with small samples with skewed data [32]. Larger randomised studies will, however, be needed to corroborate this study’s findings.

## Conclusions

The results of this single, small cost-effectiveness analysis suggest that triaging directly to physiotherapists in primary care has a very high probability of being cost-effective from a societal perspective. Also from a healthcare perspective, triaging to physiotherapists seems to lead to both economic savings and relative health gains compared to standard management.

## Abbreviations

€: Euros
CEAC: Cost-effectiveness acceptability curve
CE-plane: Cost-effectiveness plane
CI: Confidence interval
EQ5D: Euroqol 5 dimensions
GP: General practitioner
HRQoL: Health-related quality of life
ICER: Incremental cost-effectiveness ratio
MI: Multiple imputation
MSD: Musculoskeletal disorders
QALY: Quality-adjusted life-years
RCT: Randomised controlled trial
SD: Standard deviation
TAU: Treatment as usual
